# Supplementary material for: Generation of an Escherichia coli strain growing on methanol via the ribulose monophosphate cycle
Source: Nat Commun. 2022 Sep 6;13:5243. doi: 10.1038/s41467-022-32744-9 (PMC9448777; doi:10.1038/s41467-022-32744-9)
Supplement: Supplementary file 1 — Supplementary Information [file 41467_2022_32744_MOESM1_ESM.pdf]

Generation of an *Escherichia coli* strain growing on methanol *via* the ribulose monophosphate cycle

Keller *et al.*

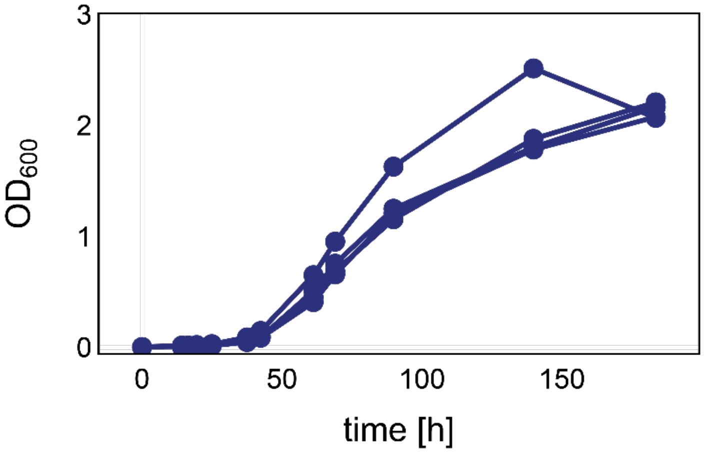

Supplementary Figure 1 Growth of single clones isolated after 534 generations. Clones were isolated from replicate 1 and 2 of the serial dilution evolution experiment. Growth was observed in minimal medium with 500 mM methanol. Source data are provided as a Source Data file.

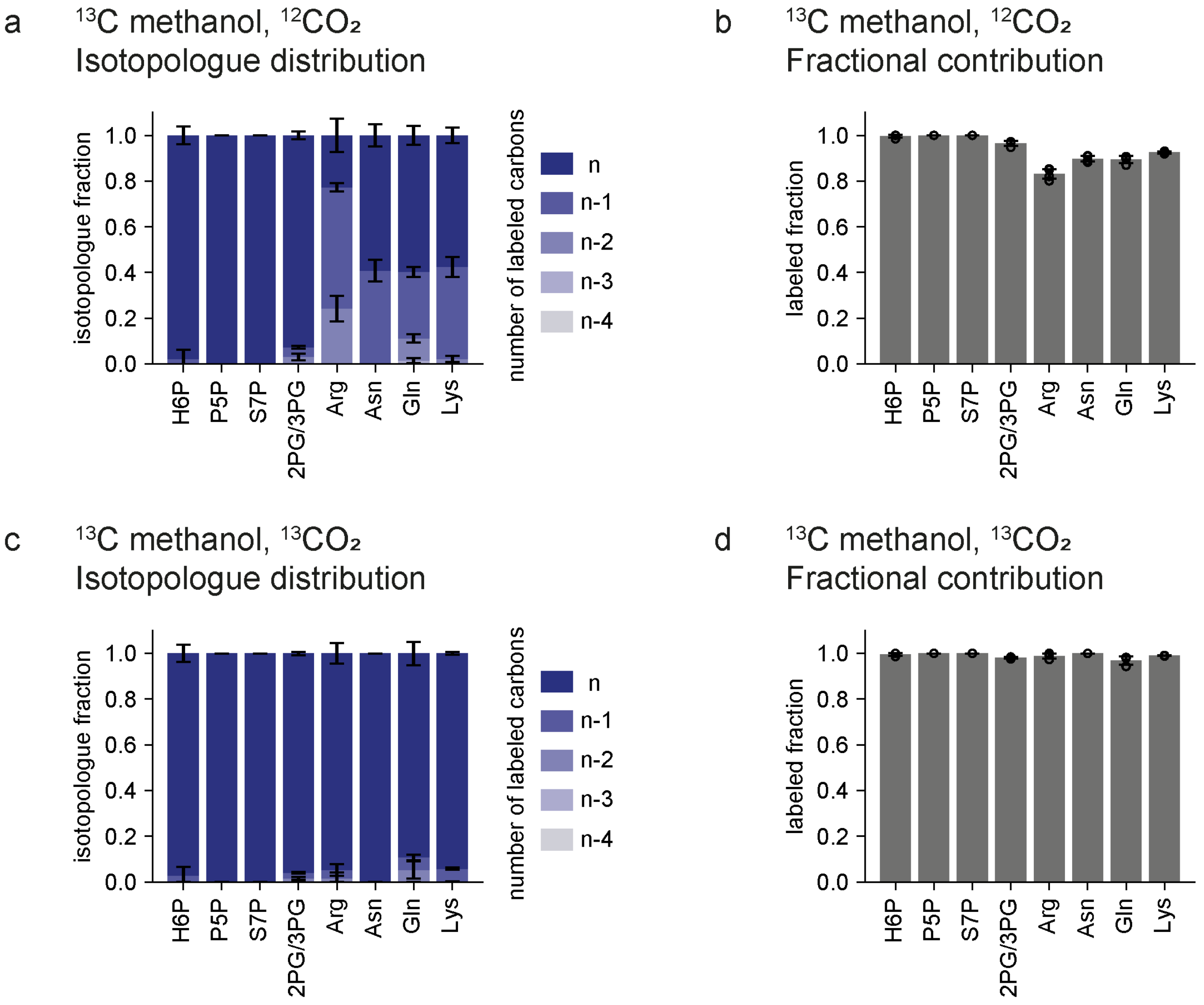

Supplementary Figure 2 Methanol incorporation into central intermediates by the evolved *E. coli* strain. Label incorporation was studied in four replicates isolated from the evolving serial dilution experiment after 384 generations. Cultures were grown in minimal medium supplemented with 500 mM ^{13}C methanol either at ambient CO_2 (**a**, **b**) or at 5% (V/V) enriched $^{13}CO_2$ atmosphere (**c**, **d**). Antibiotics, IPTG and EDTA were dropped out of the minimal medium to ensure that methanol and CO_2 are the only available carbon sources. The isotopologue distribution and fractional contribution was determined for different central metabolites (H6P, P5P, S7P, 2PG/3PG, Asn, Arg, Gln, Lys) by LC-MS. For the isotopologue distribution, n refers to the fully labeled molecule and n-1, n-2, … to molecules with one, respectively two, unlabeled carbon atoms. The different hexose phosphates (glucose 6-phosphate, fructose 6-phosphate, arabino 3-hexulose 6-phosphate), pentose phosphates (ribose 5-phosphate, ribulose 5-phosphate, xylulose 5-phosphate), and 2-phosphoglycerate and 3-phosphoglycerate were summarized as one group. S7P was detected in only 3 out of 4 replicates in the $^{12}CO_2$ condition. Data represent mean values ± standard deviation for four biological replicates for the $^{12}CO_2$ condition and for three for the $^{13}CO_2$ condition. For other metabolites, see Figure 1. Source data are provided as a Source Data file.

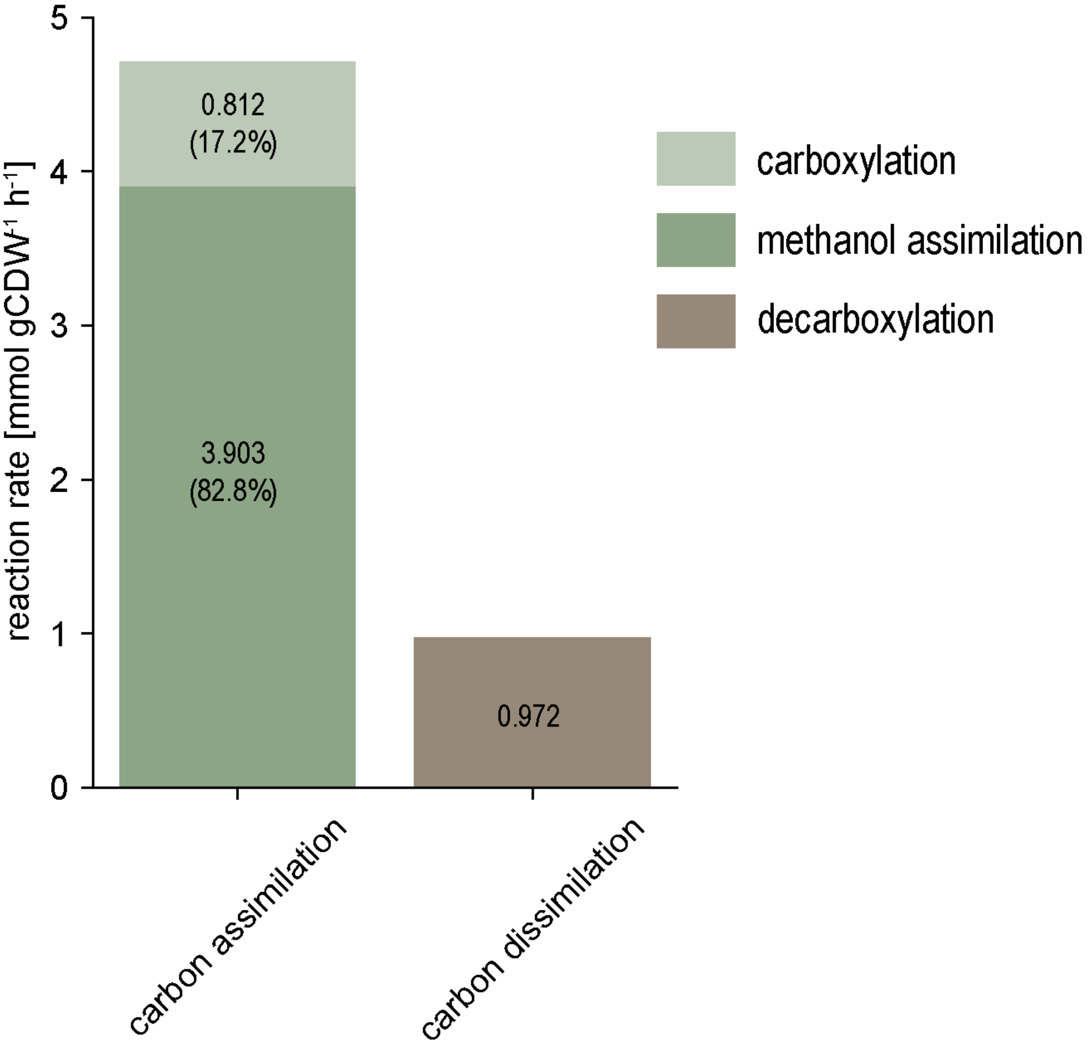

Supplementary Figure 3 Computational prediction of carbon assimilation and dissimilation reactions in the evolved *E. coli* strain using FBA. Fluxes were scaled to the growth rate of MEcoli_ref_1 (0.0856 h^{-1}) (Figure 3b). Carbon assimilation fluxes are split into two groups, methanol assimilation depicted in dark green and the sum of all carboxylation reactions depicted in light green. Carbon dissimilation, respectively the sum of all decarboxylation reactions, is depicted in brown. See Supplementary Data 6 for the corresponding reaction names and rates. Source data are provided as a Source Data file.

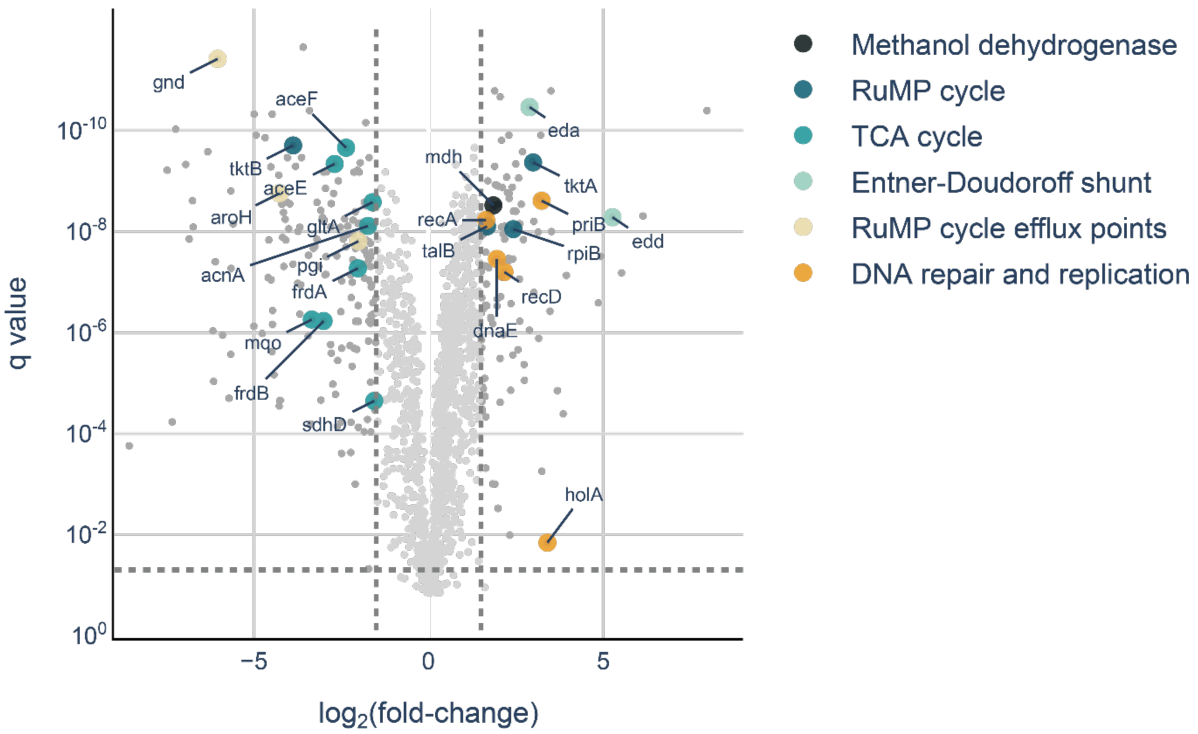

Supplementary Figure 4 Comparison of proteomes between ancestral methanol-dependent *E. coli* and methylotrophic *E. coli*. The ancestral methanol-dependent *E. coli* was grown in minimal medium supplemented with 500 mM methanol and 20 mM pyruvate. The methylotrophic *E. coli* (MEcoli_ref_1) was grown in medium containing only 500 mM methanol as carbon source. Highlighted are differentially expressed genes ($\log_2$(fold-change) ≥ 1.5) of for methylotrophy important metabolic pathways and the enriched supergroup of DNA repair and replication. The latter is a superset of the KEGG pathways mismatch repair (eco03430), DNA replication (eco03030) and homologous recombination (eco03440). Source data are provided as a Source Data file.

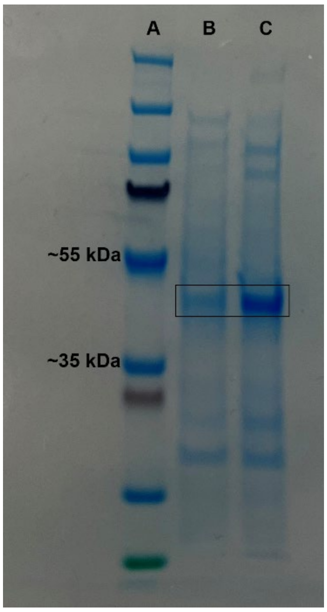

Supplementary Figure 5 Proteome comparison between ancestral methanol-dependent strain and methylotrophic MEcoli_ref_1. (A) PageRuler Prestained Ladder Plus. (B) Ancestral methanol-dependent strain grown in minimal medium supplemented with 500 mM methanol, 20 mM pyruvate to mid-exponential phase. (C) MEcoli_ref_1 grown in minimal medium supplemented with 500 mM methanol to mid-exponential phase. A marked increase in signal was observed between the two conditions for a band corresponding to a protein of equal size as methanol dehydrogenase (41 kDa). The experiment was conducted once to confirm proteomics results. Source data are provided as a Source Data file.

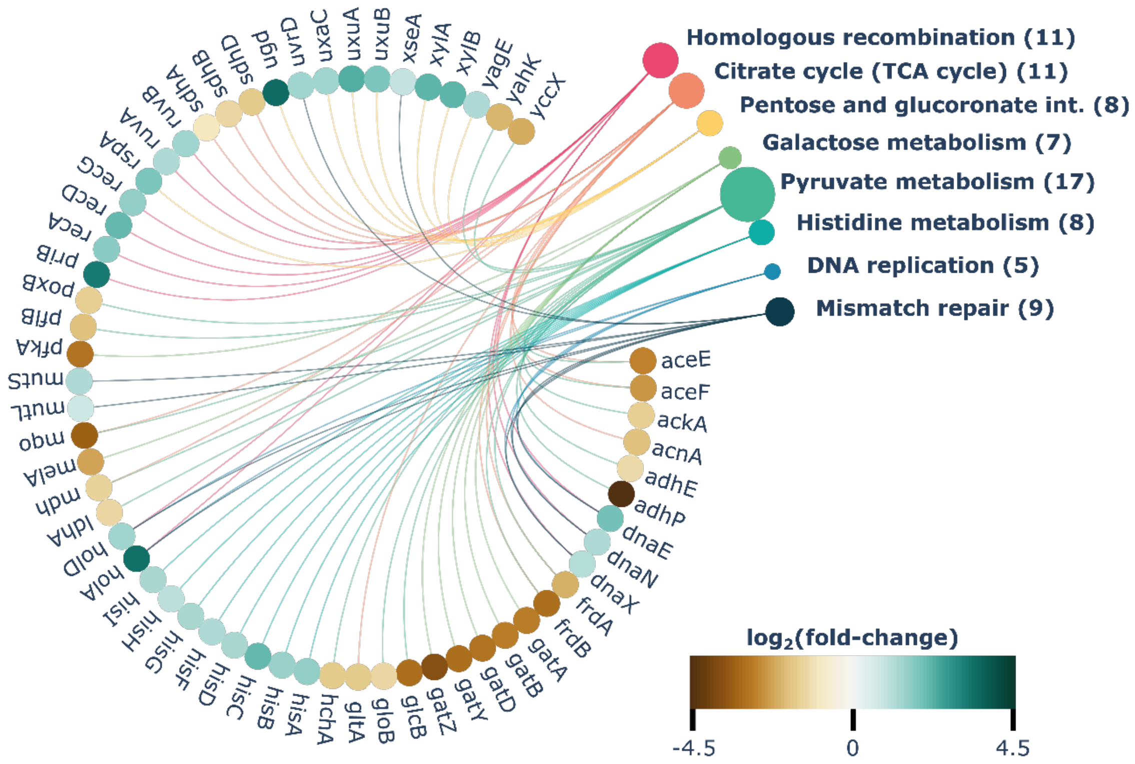

Supplementary Figure 6 Gene set enrichment analysis of proteome differences in MEcoli_ref_1 compared to ancestral strain. Eight KEGG database annotated pathways were enriched in the proteomics data and are annotated in bold. The number of associated genes is provided in parentheses after the KEGGdb pathway name and determines the size of the marker. Lines connect enriched pathways to associated genes and their expression data. Source data are provided as a Source Data file.

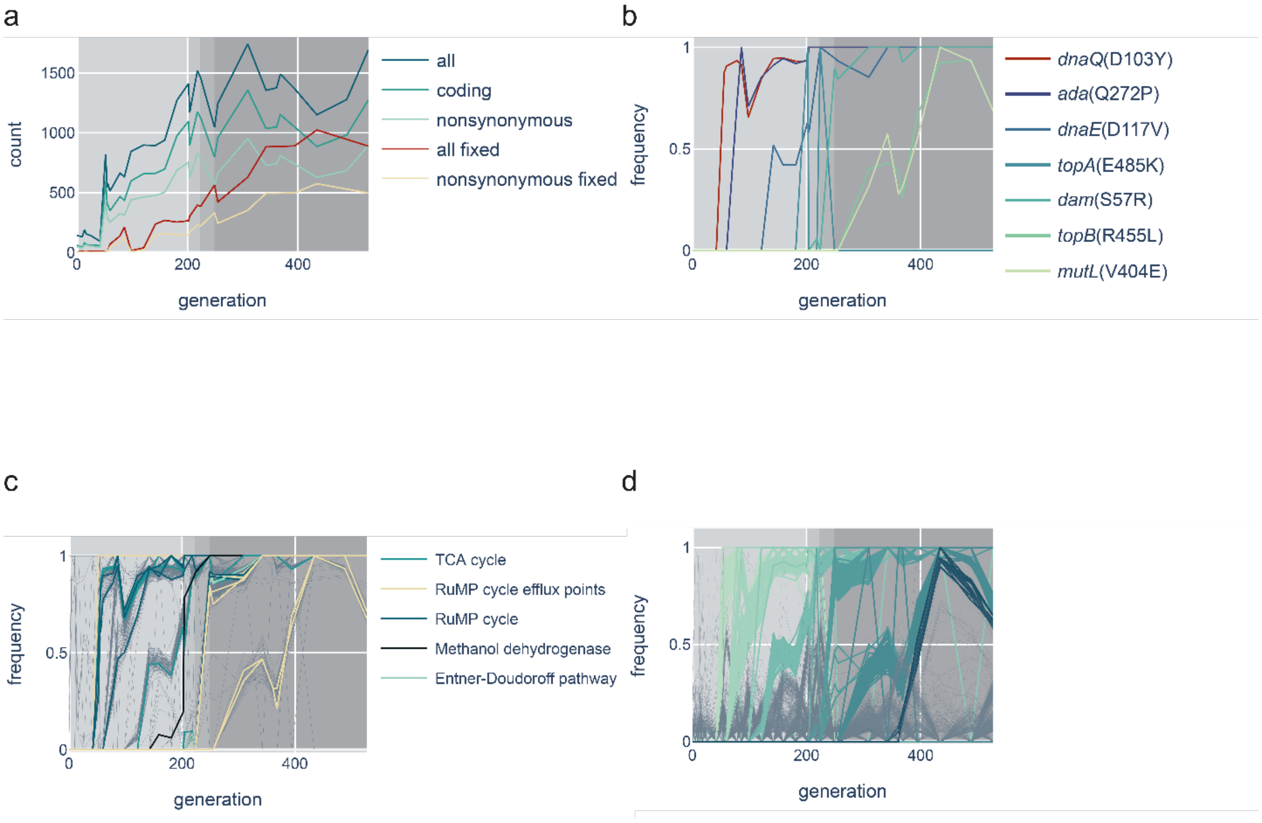

Supplementary Figure 7 Evolutionary dynamics towards methylotrophy. After methylotrophy was achieved after 249 generations, only the first serial dilution replicate lineage was sequenced at regular time intervals. From this lineage MEcoli_ref_1 was isolated. The phenotypic behavior of the other replicate lineages were similar (data not shown). **a** The number of mutations observed in the evolving population, determined by next generation sequencing, increased at a fast rate over all mutational categories. A mutation was considered fixed in the population if it was present at greater than 90% frequency in all analyses. **b** Various nonsynonymous mutations in genes associated with DNA sequence fidelity fix in the population, notably, first in *dnaQ*. **c** Nonsynonymous mutations as they fix in the population in distinct sweeps. **d** Actual population dynamics are more complex. Highlighted in different colors are distinct sweeps in the population. Additionally, competing subpopulations are present at all times (i.e. clonal interference). For example, after about 100 generations a subpopulation of different genetic makeup from the dominant strain invaded part of the population but is later outcompeted. Presumably, because the dominant strain further increased its fitness. Source data are provided as a Source Data file.

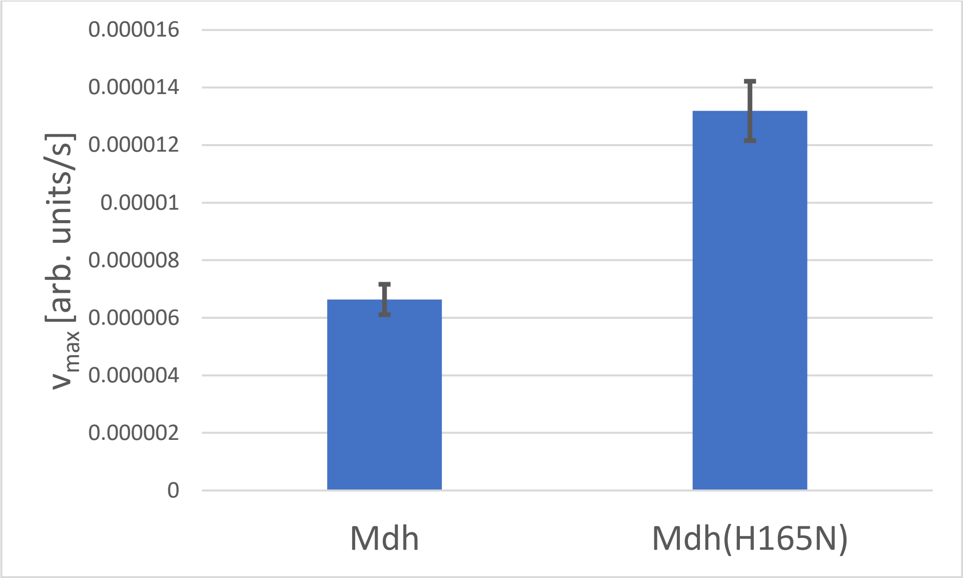

Supplementary Figure 8 Comparison of catalytic turnover numbers between ancestral and mutated methanol dehydrogenase. Maximum reaction speed was measured for purified His-tagged Mdh and Mdh(H165N). Equal amounts of protein were used in both assays and methanol turnover measured by following formation of NADH/H^+ in a spectrophotometer (i.e. absorbance increase at 340 nm over time). Data are represented as mean values ± standard deviation of six technical replicates. Source data are provided as a Source Data file.
