# Supplementary material for: Generation of an Escherichia coli strain growing on methanol via the ribulose monophosphate cycle
Source: Nat Commun. 2022 Sep 6;13:5243. doi: 10.1038/s41467-022-32744-9 (PMC9448777; doi:10.1038/s41467-022-32744-9)
Supplement: Supplementary file 3 — Description of Additional Supplementary Files [file 41467_2022_32744_MOESM3_ESM.pdf]

Description of Additional Supplementary Files

File name: Supplementary Data 1
Description: Summary of proteomics data.

File name: Supplementary Data 2
Description: List of gene symbols used in this study.

File name: Supplementary Data 3
Description: Overview of samples used for genome resequencing and associated accession numbers.

File name: Supplementary Data 4
Description: Oligonucleotides and strains used in this study.

File name: Supplementary Data 5
Description: List of reactions added to the *E. coli* core model used for flux balance analysis.

File name: Supplementary Data 6
Description: Considered reactions for evaluating carbon assimilation and dissimilation during methylotrophic growth by flux balance analysis.
